# Collective competence: Moving from individual to collaborative expertise

**DOI:** 10.1007/s40037-020-00575-3

**Published:** 2020-04-03

**Authors:** Sylvia Langlois

**Affiliations:** 1 Centre for Interprofessional Education, University of Toronto and University Health Network, Toronto, Canada; 2 Department of Occupational Science and Occupational Therapy, University of Toronto, Toronto, Canada

Individuals with multi-faceted health issues are being managed within healthcare systems that have become increasingly fragmented, often with a focus on specialization within single-disease paradigms, an approach that challenges a more holistic, patient-centred response [1–3]. Specialized profession-specific proficiency is required to deliver excellent care; however, with growing complexity comes the additional need to consider collective, team-based expertise. Yet, within education, healthcare practice settings, and professional organizations individual professional performance is often valued over collective team competence [4].

In health professions educational programs, a competency-based approach outlining desired performance and outcomes, has been gaining momentum. These profession-specific competencies, such as the CanMEDS framework outlined by the Royal College of Physicians and Surgeons of Canada [5] and a similar framework used by the Canadian Association of Occupational Therapists [6], guide education, practice, and continuing professional development in their corresponding professions. Despite the inclusion of the Collaborator role in both frameworks cited, competence in this domain is typically based on individual performance, even when measured in scenarios addressing collaborative practice (e.g. [7]). With emerging team-based assessment tools, healthcare delivery in some settings is beginning to see a shift to consideration of a collaborative focus to ensure optimal approaches and holistic care [8].

In the paper by Matus et al., published in this issue, the authors address the value of dedicated experts supporting the learning of occupational therapists who require complex capabilities in the face of challenging decision-making processes to advance and fulfill their professional roles [9]. As with many profession-specific competencies, the authors address these capabilities on the individual professional level with the intent of enhancing the provision of assessment and optimal patient care. However, extending beyond these profession-specific competencies are collective competencies, reflecting team performance needed for holistic management of patient scenarios. While not the focus of their paper, additional approaches that support team-based assessments may significantly contribute to the intended enhanced holistic response.

While individual professional competence is recognized as necessary, it is not sufficient to ensure collective competence. Collective or team competence does not simply represent a collection of individual professionals who demonstrate competency, as defined by their professional organizations. Rather, collective competence is situated within a network of complex interactions among clinicians, the patient and family members, and the organizational setting, and is markedly contextualized. In her extensive study of teams, Lingard has observed interesting characteristics of collective competence, notably that teams can still be competent when a member is incompetent, and that a team may be competent in one scenario but not necessarily in another [10]. Additionally, Lingard explores several commonly held beliefs in the world of collaborative, team-based practice [10, 11]. For example, an oft referenced approach to enhance teamwork is the belief that if clinicians have a good understanding of the roles and responsibilities of others then the team would be competent, or if all team members had a common focus on the individual patient needs, then the team would function well. Although important, this approach alone is not sufficient to create collective competence.

In exploring this notion further, we can look to Boreham and his synthesis of findings in the theory of collective competence, where he notes the following three foundational elements:

- Making collective sense of events in the workplace;
- Developing and using a collective knowledge base;
- Developing a sense of interdependency [12, p. 5].

Within healthcare, team members who work collectively to understand their specific workplace requirements, impact of organizational and government directives, as well as broader societal circumstances and determinants of health are positioned to make collective sense of their workplace environment and its expectations. The development of a collective knowledge base enhances collaboration, communication, and understanding among team members. One such example is situated in a Canadian hospital-based outpatient program, the Arthritis Program. This award-winning interprofessional program offers team-based care to individuals diagnosed with various forms of arthritis [13]. Since assessment of active joints is critical to understanding both the disease state and patient experience, the team has agreed that all members should share this common knowledge and skillset. To achieve this, they are trained collectively to assess the patient, ensuring a high degree of congruence in resulting reports. Team members still assume responsibility for their own professional expertise, but shared knowledge and assessments foster a collaborative approach. Congruently, they have an intentional focus on team dynamics and shared working relationships among members, resulting in offering an exceptional patient-centred program. Finally, a sense of interdependency regarding each other’s roles and responsibilities is critical. Choices and recommendations made by one professional on the team may have an impact on the decision-making process of other clinicians, and ultimately on the patient receiving care.

Guidance on considering team processes can be found in interprofessional competency frameworks. Although there are several international examples, I will reference the one developed by the Canadian Interprofessional Health Collaborative [14]. The following dimensions of Interprofessionalism are described as: Role Clarification, Interprofessional Conflict Resolution, Team Functioning, Interprofessional Communication, Patient/Client/Family/Community-Centred Care, and Collaborative Leadership; each serves as a guide for teams to support growth in how members interact with each other. Team-based critical thinking, problem-solving, and reflection enable collective development of these dimensions. In practice settings, as described in the earlier scenario, the balance of explicit attention to team learning of task-oriented, clinically relevant, patient-focused content, as well as process, including interactions and team dynamics as determined by the interprofessional competencies described, will enable team-based learning and optimal performance. In a growing number of educational settings, a focus on collaborative competency development of health profession students, participating in interprofessional education curricula, addresses both individual and team-based skills; an example is the University of Toronto Interprofessional Education curriculum [15]. Although assessment of collaborative competencies is anchored in individual acquisition, collective competence is measured when students work together for an extended time, enabling them to translate the practice to future work settings.

In conclusion, individual competency in profession-specific tasks is indeed a critical component of effective healthcare practice; however, it must be enacted within the context of collective team-based competency. To develop these collective competencies, teams must explicitly address both expertise relevant to the area of practice *and* process components that address how they function collectively. Team-based learning in the workplace will foster pertinent skills and tasks required for holistic, patient-centred interventions and management, enabling the important cultural shift that embraces individual as well as collective competence.
